# Consumers’ Trust in Different Sources of Information Related to Food Hazards and Their Judgment of Government Performance—A Cross-Sectional Study in Brazil

**DOI:** 10.3390/foods12173285

**Published:** 2023-09-01

**Authors:** Peter Rembischevski, Eloisa Dutra Caldas

**Affiliations:** 1 Brazilian Health Regulatory Agency (Anvisa), Brasilia 71205-050, DF, Brazil; peter.rembischevski@anvisa.gov.br; 2 Laboratory of Toxicology, Department of Pharmacy, Faculty of Health Sciences, University of Brasilia, Darcy Ribeiro Campus, Brasilia 70910-900, DF, Brazil

**Keywords:** food hazards, government performance, trust in information sources, pesticides, genetically modified food

## Abstract

Trust in institutions is fundamental for the stability and proper functioning of democracies, particularly in matters of high public sensitivity, such as food safety. This study aimed to assess trust levels in different sources of information and respondents’ evaluation of the performance of government agencies responsible for controlling food-related hazards. Individuals interviewed in three environments (hospitals/clinics, supermarkets, universities, *N* = 1000) answered a face-to-face questionnaire in the Federal District of Brazil, and another population (health surveillance employees at the municipal, state and federal levels; *N* = 1017) answered the questionnaire online. About 60% of the population interviewed considered government performance to be low/very low. Scientists/universities, medical doctors (MD)/health professionals, and nongovernmental organizations (NGOs) were judged to be the most reliable sources of information on food hazards, while the food industry, supermarkets and social media inspired the lowest trust. Individuals from the hospitals/clinics group had significantly higher trust in MD/health professionals, media and websites than the two other Federal District groups. In general, income and education were the most predictive factors for the results, being negatively associated with assessment of government performance and trust in most information sources. In the Federal District, there was a negative association between trust levels in the government and worry about pesticides and genetically modified food, but a positive association between trust in NGOs and worry on these hazards. The results point to the need for the implementation of more effective communication strategies by institutions in which the population has low trust levels, such as government and food companies.

## 1. Introduction

Social trust can be defined as a belief in individuals’ and institutions’ honesty, integrity and reliability. In a broader sense, trust refers to the belief that others will act as expected of them [1]. Trust in others’ actions (whether people or institutions) is one of the foundations for community life in society, and the key to social and economic development and to individuals’ well-being [2].

Negative events have a greater impact on self-reported trust than positive ones [3], and individuals tend to trust more those who share their values, beliefs and attitudes towards a given topic, in light of the concepts known as salience and similarity of values [4]. In the absence of laypeople’s knowledge, particularly on technically complex and/or controversial issues, trust in organizations is crucial for their decision-making and the ways in which they try to reduce the system’s complexity [5,6]. On the other hand, when knowledge about a topic is high, the importance of trust for risk perception is substantially reduced. In other words, the impact of social trust on risk perception may be mediated by individuals’ knowledge [5]. It is also important to highlight that, although performance and trust are different constructs, perception of institutions’ performance is strongly correlated with trust in them [7]. Sources of information regarding food-related issues include scientists, health professionals, government, and conventional and social media, with the first two inspiring the highest trust level and social media among the lowest [8,9,10,11,12,13]. Furthermore, age, gender and social class may have an impact on trust in some information sources, although the results depend on various factors, including the focus and the design of the study [12,13,14,15,16]. Government authorities should be aware of the population’s level of trust in their actions and target specific groups to improve risk communication on food safety issues.

There is a large set of evidence showing that trust in risk regulation is strongly related to the perception and acceptability of risk, although it is unclear whether trust is a consequence or a cause of risk perception [17,18]. An inverse correlation between perception of the risk of genetically modified (GM) foods and trust in the institutions that regulate them among the British population was reported by Poortinga and Pidgeon [19]; this correlation was strongly reduced when the analysis was controlled for the acceptability of GM foods. In Italy, Rosati and Saba [8] assessed the risk perception associated with different food-related hazards (including GM food and pesticides) and trust in different sources of information. Consumer associations, research institutes and nongovernmental organizations (NGO) had the highest trust scores and also were perceived as the most knowledgeable about food-related risks, the most concerned about citizens’ safety, and the most honest in terms of completeness of information. On the other hand, the government was perceived as the least trustworthy and honest. More recently, a national survey conducted in China showed that respondents with low food risk perception have high levels of trust in government competence, benevolence and honesty, while highly educated people trust the government less [20].

Although some studies have evaluated social trust in information sources and government performance in Latin America, including Brazil, none have focused on issues related to food safety [21,22,23,24]. The present study aimed to assess levels of trust in different sources of information and assessment of the performance of government agencies responsible for controlling food-related hazards among individuals interviewed in three different environments in the Federal District of Brazil, through a face-to-face questionnaire, and among Brazilian health surveillance employees through an online questionnaire, considering their sociodemographic characteristics. Additionally, the association between trust in the government and in NGOs and concern about heavy metals, pesticides and GM foods was investigated. To the best of our knowledge, this was the first study to evaluate the trust of the Brazilian population in relation to different information sources regarding chemical risks in food and its relationship with risk perception in food hazards. Furthermore, the investigation of the impact of the interview environment on trust in information sources regarding food-related issues is also novel.

## 2. Data Collection and Methods

### 2.1. Questionnaire

The questionnaire applied to the participants contained 21 food-related questions in addition to sociodemographic questions (Supplementary Materials) and was part of a broader project involving risk perception issues ([25], questions 6, 7, 14, 15, 21, 22), food-related attitudes ([26], questions 3–5, 8–13, 16–18), and assessment of government agencies and trust in information sources related to food risks, which is the focus of this paper (questions 19, 20, 23). The questionnaire was previously tested with a group of 20 individuals from the general population for final adjustments. The project was approved by the Research Ethics Committee of the Faculty of Health Sciences, University of Brasilia (71667117.5.0000.0030), and all participants signed an informed consent form.

The questions that are relevant for this study (Supplementary Materials) are: (19) how the performance of government agencies responsible for ensuring food chemical safety is judged (very good/good, acceptable, low/very low or no opinion); (20) the preferred actions to improve this performance (increase inspection in industries, increase inspection in commerce, apply more fines to offenders, improve risk communication, prioritize consumer health, require more information on the food label); and (23) the level of trust in the following information sources when conveying correct information about the risks arising from the presence of chemicals in food: media, websites/blogs, social media, scientists/universities, government authorities, nongovernmental organizations (NGOs), food industry, supermarkets, medical doctors (MD)/health professionals, farmers and family/friends. Responses to the last question were given on a three-level scoring scale: low/don’t trust; trust reasonably; and trust a lot. The option “don’t know/don’t have an opinion” was not scored. Reliability (internal consistency) of the three questions within the questionnaire was scored at a Cronbach’s alpha value of 0.70, considered acceptable.

#### 2.1.1. Federal District Population

The questionnaire was administered from May 2018 to December 2019 through face-to-face interviews with 1000 individuals in three different environments in the Federal District of Brazil: (1) supermarkets (*N* = 400); (2) public and private universities (*N* = 300, only students); and (3) outpatients in public hospitals and private clinics (*N* = 300). The study used a convenience sampling approach until the pre-established number of interviewees was reached. Only individuals 18 years old or over, literate, and with no serious intellectual or physical impairment participated in the study and signed the informed consent form. Most participants (57.8%) were women, 46.7% were between 18 and 30 years old, 55.5% had incomplete or complete college education, and 49.8% had a household income between 2 and 10 times the minimum wage (MW), which at the end of 2019 was about USD 250. A summary of the sociodemographic information of all participants is shown in Table 1, and information for each group is described elsewhere [25].

#### 2.1.2. Health Surveillance Employees in Brazil (Health Employee Population)

To reach health surveillance professionals around the country, the same questionnaire was administered online to employees of the Brazilian Health Regulatory Agency (ANVISA) and of municipal and state Sanitary Surveillance Departments (SSD), using a platform developed by the Brazilian Ministry of Health (FormSus). An email with the link to the questionnaire was sent to about 1500 ANVISA employees in July 2019 and to about 6200 SSD employees in December 2019, remaining available for 45 days in both cases. In total, 1017 employees answered the online questionnaire, of which 66.8% were women, 60.5% were 31 to 49 years old, 29.4% had a family income between 5 and 10 MW, and 62.7% completed graduate school (Table 1).

### 2.2. Statistical Analysis

Data from the questionnaires were input into Epi Info™ 7.2.2.6, and statistical analysis was performed in IBM SPSS Statistics V.28. Ordinal logistic regression analyses were performed to test the impact (main effects) of belonging to a group and of sociodemographic parameters (gender, age, income, and education) on dependent variables (assessment of government performance and trust in the information sources). The parameters of age, education, and family income were categorized as follows. Age: up to 24 years, from 25 to 49 years, or 50 years and older in the Federal District population, and from 18 to 30, 31 to 49, or > 50 years for the Brazilian health employee population. Education: up to high school or university or more. Family income: up to five MW or above five MW. Results are given in odds ratio (OR) [lower level-upper level at 95% confidence level], *p*.

No variables overlapped in the adjusted model (variance inflation factor lower than 4 and tolerance higher than 0.1). The main assumption of the model based on ordinal regression (proportional odds) was checked by the parallel lines test, and all analyses indicated that the model was appropriate to explain the results, except trust in farmers in the Federal District group and trust in media in the health group. In these two cases, the analysis was performed by multinomial logistic regression. For all analyses, the impact of each independent variable was first tested separately (bivariate analysis), and those that showed significance were included in the final adjusted model (multivariate). Risk perceptions regarding GM food, heavy metals and pesticides for the Federal District population had been assessed previously [25], and their association with trust in government and in NGOs as information sources was investigated in the present study using binomial logistic regression.

## 3. Results

### 3.1. Evaluation of Performance of Government Agencies and Trust in Information Sources Related to Chemicals in Food Reported by the Federal District Population

About 60% of the population interviewed in the Federal District judged the performance of government agencies responsible for controlling chemical food hazards in Brazil as low/very low, 20–21% found it acceptable and between 8 and 12.3% considered this performance good/very good; 10% of participants declined to comment. The results of the ordinal regression analysis indicated that individuals from the supermarket group (related to the hospitals/clinics) and those with a lower education level were significantly more likely to judge the performance as better, compared to their counterparts (OR = 1.49 [1.04–2.12], *p* = 0.028, and OR = 1.85 [1.29–2.66], *p* = 0.000, respectively).

**Figure 1:**
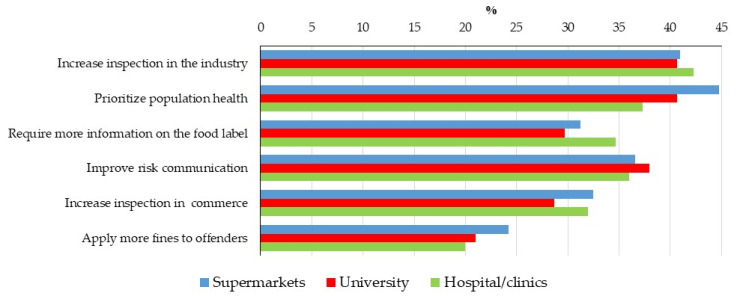
Necessary actions to improve governmental performance to guarantee food safety, in percentage of the individuals that assessed the performance in each Federal District group as low/very low (57.6 to 61.7% of the participants).

Among those individuals who indicated low/very low governmental performance, the actions deemed necessary to improve it were well distributed among the three groups (Figure 1). The most highlighted options were “increase inspection in industries” (40 to 42%) and “prioritize the population’s health” (37.3 to 44.7%).

**Figure 2:**
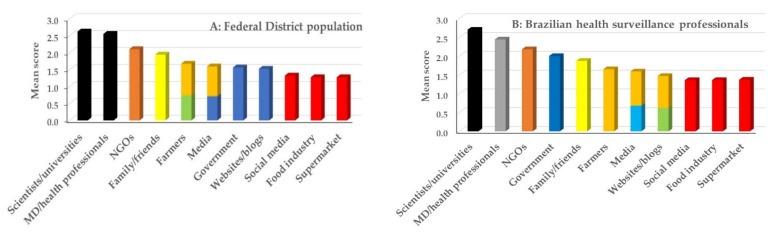
Mean score of trust in different information sources related to the chemical hazards in food in (**A**): respondents in the Federal District and (**B**): Brazilian health surveillance professionals. Different bar colors indicate that the mean scores are significantly different (*p* < 0.05). Kruskal–Wallis test followed by Dunn’s. MD = medical doctor; NGOs = nongovernmental organizations.

Figure 2A shows the graphical representation of mean scores related to the information sources within the Federal District population, with different bar colors indicating significant differences between the scores (*p* < 0.05). Trust scores for scientists/universities and medical doctors/health professionals were higher (~2.5), while social media, food industry, and supermarkets scored the lowest among all sources of information (~1.3). Trust in the conventional mass media received similar scores to farmers, government and websites (~1.5; *p* > 0.05).

Table 2 shows the result of ordinal or multinomial logistic analysis of trust in relation to the group and sociodemographic parameters for the Federal District population. Government authorities and the food industry were not impacted by any variable in this approach and are not shown in the table. Belonging to a group significantly (*p* < 0.05) affected trust in MD/health professionals, media, websites and social media, with hospitals/clinics and/or supermarket groups having significantly more trust in these sources than students in universities (OR = 1.63 to 2.13). Older individuals showed less trust in scientists/universities, MD/health professionals, and websites than those 24 years old or younger (OR = 0.450 to 0.590), while trusting more in family/friends (OR = 2.04 and 3.09). Gender only affected trust in NGOs, with women trusting more than men (OR = 1.53). Individuals with lower household incomes (<5 MW) had more trust in farmers, media, social media and websites (OR = 1.37 to 1.71), while those with lower education levels (up to high school) trusted more in family/friends, farmers and supermarkets (OR = 1.96 to 3.21), but less in scientists/universities (OR = 0.682).

### 3.2. Association between the Trust and Risk Perception Variables among the Federal District Population

Possible associations were tested between trust in government agencies and NGOs and interviewees’ worry about pesticides, GM food and heavy metals. These three hazards presented the highest score for concern among the 11 hazards investigated in the previous study [25]. As for trust items, the government and NGOs were selected for their positions of constant antagonism in society. The results are shown in Table 3.

A negative correlation was observed between trust in the government and worry about the presence of pesticides and GM food (OR = 0.694 and 0.503), i.e., individuals with high concern for these hazards trust the government less, and vice versa. On the other hand, the association was positive for heavy metals (OR = 2.1). Furthermore, individuals with high concern regarding pesticides, GM food and heavy metals reported high trust in NGOs (OR = 2.21 to 6.48).

### 3.3. Evaluation of Performance of Government Agencies and Trust in Information Sources among Brazilian Health Surveillance Employees

The assessment of government performance by Brazilian health surveillance employees was similar to that of the Federal District population (60% considered it low/very low, 20% acceptable and 14% good/very good).

Figure 2B shows the mean trust scores that health surveillance professionals reported for the different information sources. The profile is mostly similar to that of the Federal District population (Figure 2A), with scientists/universities, MD/health professionals and NGOs being the most trusted sources, and supermarkets, social media and food industry the least trustworthy. The major difference was the position of trust in the government, which was placed fourth in the ranking, compared with ranking seventh in the Federal District population (mean scores of 2 and 1.6, respectively). Indeed, a higher percentage of health surveillance employees reported medium/high trust in government (71% of the population) compared with the Federal District population (47.6%). This comparison was similar for the other information sources.

Table 4 shows the results of ordinal or multinomial logistic regression for information sources according to the sociodemographic characteristics of the health surveillance population. NGOs and the food industry were not significantly affected by any variable. Individuals 50 years or over trusted significantly less in scientists/universities and media than those from 18 to 30 years of age (OR = 0.515 and 0.300), and the older group trusted more in government than younger respondents (OR = 1.45). Individuals 31 years and over trust significantly more in family/friends (OR = 3.46 and 1.47), while women trust the media less than men (OR = 0.479), making the media the only source of information significantly impacted by gender. Individuals with lower income and/or education trust significantly more in MD/health professionals, family/friends, farmers, media, websites, social media and supermarkets (OR = 1.62 to 2.17). On the other hand, individuals with lower income and less education trust significantly less in the government and scientists/universities, respectively (OR = 0.684 and 0.398).

## 4. Discussion

This study investigated how two Brazilian populations judge the performance of governmental institutions responsible for ensuring food safety, and examined these populations’ trust in different sources that provide information concerning the potential risk present in food. The questionnaire was answered by individuals in different environments in the Federal District (universities, supermarkets and hospitals/clinics), and online by Brazilian health surveillance employees at the municipal, state and federal levels. Convenience sampling, a non-probability and nonrandom technique where no extrapolation for the whole population can be made, was used in the Federal District [27]. The profile of this study population is similar to that in the region regarding gender and income [28]. Most of the population is female (52.2% vs. 57.8% in the study), and less than 10% has a family income under or equal to USD 250 (4.4% vs. 9.7%), with 19–26% in each of the other income categories. However, the Federal District population age in this study is younger than the regional population (16.5% vs. 46.7% were 18 to 30 years old), mainly because 94.3% of students in the universities were 30 years old or younger [25].

The performance of government institutions was considered low or very low by about 60% of both populations. Although this result may be expected for the Federal District population, which includes individuals from different occupations, it was unexpected for the health employees. It is possible that the employees exercise self-criticism and/or pass judgment on governmental agencies other than their own. This last hypothesis probably had more weight in the answers, as some employees explicitly commented in the on-line questionnaire about other control agencies that were performing poorly. Furthermore, municipal and state health surveillance employees may feel powerless to carry out what, in their view, is better control of chemical substances and technologies in food, since the federal government level (Anvisa) must regulate and establish acceptable/tolerable limits for residues and contaminants. It should be noted that in terms of trust in government as a source of information, health employees showed a higher mean score than the Federal District population (2 vs. 1.6), indicating that it is possible to trust the institutions and at the same time judge that their performance is poor or could be better.

Both populations showed significantly higher trust scores in scientists/universities, MD/health professionals, and NGOs compared with the other information sources, a finding that agrees with studies conducted elsewhere [9,29]. Unlike the Federal District population, health employees showed a significantly higher score for scientists/universities than MD/health professionals (2.7 vs. 2.4), which may be explained by the fact that a much larger proportion of this population attended graduate school (62.7 vs. 15.1%). The 2019 Eurobarometer showed that most Europeans are likely to trust scientists and consumer organizations (~80%) for information on food-related risks, but trust NGOs less (56% [29]). In Australia, a study conducted in 2011 indicated that over 90% of the 298 participants were confident/extremely confident about the food product information they receive from health professionals, scientists and food regulators [9].

In the present study, the trust score regarding information sources was similar for the government and the media in the Federal District, but significantly higher for the government among the surveillance health employees, which was expected, as discussed earlier. Trust in government agencies does not seem to be related to media coverage, which most often presents a negative interpretation of events involving food-related hazards and the allegedly poor control by Brazilian government authorities. The fact that about 30% of the population in this study considered the performance of government agencies to be at least acceptable can, in a superficial analysis, be interpreted as satisfactory, but is much lower than the levels measured by the 2019 Eurobarometer, which showed that 60% of respondents trusted in national and EU authorities [29].

In a study conducted with 1000 respondents in Italy using a five-point scale, Rosati & Saba [8] observed higher scores of trust assigned to research institutions and consumer associations (higher than 4), and lower scores for government institutions, producer associations and reporters, in line with the results of the present work. Frewer et al. [30] observed that knowledge alone is a poor predictor of trust, and that institutions that have some accountability, a term that also encompasses transparency and responsibility, are the sources seen as the most reliable. Except for the level of trust in the government, trust in the other information sources differed little between the populations of the Federal District and health surveillance employees. The psychometric paradigm stipulates that the lay public perceives risks in general, but particularly those of technological origin, more sharply than experts in the field [31]. Such reasoning could be extended to trust levels, since they are related to perception and it is known that greater knowledge reduces dependence on other parties regarding trust [5]. Starting from the premise that health surveillance employees are specialists (even those in administrative areas, as they are within a technical environment) and the Federal District interviewees are laypeople, in principle, the results of this study suggest that the knowledge gap does not seem to have a substantial impact on trust levels in information sources.

In both populations, supermarkets and the food industry scored significantly less for trust than the other information sources. In Europe and Australia, supermarkets and/or food industries are also among the least trusted information sources for food-related issues [9,29]. It is possible that their for-profit characteristics generate the perception of conflict of interest and cause skepticism in the population. Additionally, low trust may also be due to the lack of information about the food manufacturing process, which is usually considered intellectual property and is not disclosed by companies [32]. About 69% of Europeans trust farmers to convey information about food risks [29], a higher percentage than in the present study, in which about 50% of respondents trust this group at least somewhat. Jonge et al. [33] found that consumer confidence in the safety of food is strongly enhanced by trust in food manufacturers, more than trust in the government, farmers, and retailers, probably because manufacturers are perceived to have more responsibility for the safety of food. The authors also concluded that the importance of different actors and trust levels leading to confidence may depend on the type of food.

Social media is among the information sources with the lowest trust score in both studied populations, similar to what was found in Australia, where only 11% of the participants trusted this source for issues related to food products [9]. Indeed, social media has been increasingly recognized as a source of unreliable information, exemplified by the phenomenon of echo chambers, which are groups of like-minded users framing and reinforcing a common narrative [34], and of fake news, in what has already been called the post-truth era [35]. In both populations, trust in social media was lower than in mass media (television, radio and newspapers), but comparable to that in websites. Mass media and the internet were the information sources Italians use most when searching for information about food safety, although they are highly distrusted compared to other sources [10]. Although contradictory, this trend was also reported by Omari et al. [36] in a study conducted in Ghana.

In general, trust in institutions is lower in countries with greater social inequality. Nordic countries’ institutions enjoy the highest trust levels in the world, while levels are persistently low in South America and other developing countries, and the USA and Western European countries are in an intermediate position [21,37]. According to the 2022 Edelman Trust Barometer, trust in the Brazilian government dropped from 65% in 2020 to 52% in 2022, when 60–64% of the Brazilian population trusted in businesses and NGOs; a similar trend was found at a global level [22].

In the Federal District, individuals were interviewed in hospitals/clinics, supermarkets and universities (only students), and the focus was the environment, as the participant could potentially be in any environment at another time (nobody responded to the questionnaire twice). Although the sociodemographic profile between the groups was significantly different regarding age (younger individuals in the university group) and income/education (lower in the hospitals/clinics group) [25], this profile is adjusted in the logistic regression model. The impact of the environment on trust in information sources on food-related issues was more significant for the hospitals/clinics group. Compared to the university group, the individuals from this group trust MD/health professionals significantly more compared to the university group, which may be due to the healthcare environment; the hospitals/clinics group also trusted more in the media and websites. On the other hand, the university group showed a significantly lower trust in social media and websites compared with the supermarket group. Although they are the youngest population in the Federal District [25], and most likely use social media more than older individuals [16], the university environment may bring a more critical view about the origin of information.

The impact of age may be different when the trust is interpersonal (e.g., between family and friends) or institutional [2]. Indeed, in the present study, older individuals trusted significantly less in scientists/university, MD/health professionals and/or websites, while they trusted more in family/friends and the government (only the Brazilian surveillance employees). Li and Funk [16] also found a positive correlation between age and trust in family/friends across 38 countries. Studies on social trust between genders have produced mixed results or no significant differences [15,38]. In a study conducted in the Czech Republic, men and older individuals had a higher level of trust in social media [11], an association not found in the present study. McDermott and Jones [15] have demonstrated that a feminine personality contributes to higher levels of trust in the American government, as women are more communal and more likely to have faith in government actions. In the present study, however, gender significantly impacted trust for only one of the eleven information sources in each population studied (greater trust in NGOs among women from the Federal District and less trust in the media among female health employees). The previous study conducted with the same Federal District population has shown that women have higher risk perception regarding most food hazards [25], which may have an impact on higher trust in NGOs, which are involved in activities such as consumer protection and environmentalism.

Studies that investigate the relationship between education/income and trust in government agencies and other institutions show different findings, as they are mediated by several other factors, including data sources, the samples, and the methods applied [39,40]. As people’s education levels increase, their expectations regarding government performance increase, and if their expectations grow faster than the government’s actual or expected performance, trust tends to decline [39,40]. A hypothesis can be drawn that individuals with lower income/education have lower expectations in life, relying more on the information they receive from others, and having less critical judgment in that regard. Bálint and Boda showed a strong and significant correlation (r = 0.817, *p* < 0.001) between economic development and institutional trust in European countries [39], while highly educated Chinese people have low levels of trust in government competence [20]. In the present study, no association was found between trust in government as an information source on food-related hazards and education or income in the Federal District, but income had a negative association among Brazilian health employees. However, income and/or education were the most predictive variables for the trust construct related to most of the other sources of information, with a negative association, the exception being trust in scientists/universities, which was higher among individuals with a higher education level, as could be expected. The inverse association between education and trust in the information provided by family/friends is intuitive. A higher education level gives the individual the security to trust his/her own knowledge and means of obtaining the desired information.

Rembischevski et al. [25] showed that heavy metal and pesticides had the highest risk perception scores within the Federal District population, while GM food ranked 10th among the eleven hazards included in the survey. The negative association between trust in government agencies and risk perception regarding pesticides and GM foods found in this study was also reported by Siegrist & Cvetkovich [5] among 91 university students in the USA, in Britain [10], and in Italy [11]. In a national survey conducted in China, Han and Yan [20] concluded that reducing the public perception of food risk is a way to enhance trust in the government.

The positive association between trust in NGOs and risk perception regarding pesticides and GM goods seems intuitive and is corroborated by the concept of the affect heuristic [41], given that these organizations constantly criticize government regulatory agencies. In other words, high risk perception regarding chemical/technological agents present in and/or related to food is inversely associated with confidence in those who are responsible for controlling their risks, but positively associated with trust in institutions that oppose government agencies and call for more rigorous controls on these risks.

The positive and similar association between risk perception regarding heavy metals and trust in government agencies and NGOs indicates some ambiguity, and seems inconsistent with the results for pesticides and GM foods. Heavy metals are ubiquitous in nature, but their levels can be higher in food grown in areas close to mining and metallurgy sites [42]. Although they had the highest risk perception among all the items studied by Rembischevski et al. [25], it seems that individuals trust the information they receive from the government and recognize its efforts to keep contamination levels as low as possible.

In China, those most concerned with food safety trust physicians and research institutes (scientists) more, regardless of their knowledge level. The expertise of information sources, as well as their honesty and the demonstration that they care about people, were positively correlated with trust [43].

According to Verbeck et al. [44], traceability and labelling, segmented communication approaches and public involvement in risk management are possible strategies to restore consumer confidence in decision-makers. In a study conducted in a Brazilian city, most of the 224 participants (74.6%) did not recognize the symbol used in the mandatory labeling for GM foods, and many who identified the symbol had difficulty in interpreting it [45]. The authors found that risk perception was moderated by social trust and perceived quality, and consumers with a high confidence in science and the government had reduced risk perception regarding GM foods.

### Limitations

The main limitations of this work concern the accuracy of some responses, considering the possibilities of bias and factors such as haste or fatigue of respondents when answering the questionnaire, and the order of the questions within the questionnaire. Another limiting factor is that the trust score had only three levels, in contrast to the more usual five or seven-point Likert scale, which could have decreased the sensitivity of the test. Finally, it is important to note that the study conducted in the Federal District involved convenience sampling, and the results may not be representative of the Federal District population. The sampling limitations of the online questionnaire answered by health surveillance employees may be lower, as a much larger universe was invited to participate in the research. However, in both methods, an important bias concern is that people who were willing to participate in the study are probably more interested in and sensitive to the topic than those who decided not to participate. It is also worth mentioning that the comparisons made between the results of the Federal District population and health surveillance employees must be interpreted with caution, as they comprise different territorial scopes, and different questionnaire application methods were used (face-to-face vs. online).

## 5. Conclusions

In this study, scientists, health professionals and NGOs were the most trusted information sources on food-related issues, as reported by the participants. Although Brazilian health surveillance employees ranked trust in the government higher than the Federal District population, most of both populations judged the performance of Brazilian government agencies responsible for guaranteeing food safety to be low. Furthermore, individuals with high risk perception about pesticides and GM foods had lower trust in the government. In general, older individuals trusted more in most of the information sources, as did those with lower income/education. Additionally, individuals in the hospitals/clinics group trusted more in health professionals than the other groups, which may be linked to the environment they were in during the interview.

To have their functions recognized and to be seen by the population as reliable in guaranteeing food safety, institutions involved in food production and regulation need to continually increase the transparency of their actions. Therefore, it is necessary to leave aside the formal approach that has been the keynote of the main Brazilian governmental institutions, and move to a communicative approach based on exchange, dialogue, and interaction with other individuals and with society in general, targeting specific population groups.

## Figures and Tables

**Table 1:** Sociodemographic characteristics of the studied populations in Brazil, *N* (%).

Characteristic	Federal District, *N* = 1000	Health Employees, *N* = 1017
Gender		
Female	573 (57.8)	678 (66.8)
Male	414 (41.8)	337 (33.2)
No response/others	14 (1.4)	2 (0.20)
Age range, years		
18–30	462 (46.7)	138 (14.1)
31–49	310 (31.3)	593 (60.5)
50–65	182 (18.4)	239 (24.4)
>65	36 (3.6)	10 (1.0)
No response	11 (1.1)	37 (3.6)
Family income, MW		
Up to 1	95 (9.7)	32 (3.2)
>1 to 2	186 (18.6)	111 (11.0)
2 to 5	254 (26.0)	287 (28.6)
5 to 10	238 (23.8)	295 (29.4)
>10	202 (20.2)	280 (27.9)
No response	26 (2.6)	12 (1.2)
Education		
Primary school, including incomplete	76 (7.6)	3 (0.21)
High school, incomplete	31 (3.1)	6 (0.59)
High school	188 (18.8)	68 (6.74)
College, incomplete	354 (35.4)	61 (6.0)
College	201 (20.1)	218 (21.6)
Graduate school	151 (15.1)	633 (62.7)
No response	0 (0)	8 (0.79)

MW = minimum wage (USD 250).

**Table 2:** Ordinal or multinomial logistic regression analysis (for farmers only) of the trust in different sources of information, by population group and sociodemographic characteristics of the Federal District population. ^a^ Results are given as odds ratio (*p*).

Variable		Scientists/ Universities	MD/Health Professionals	NGOs	Family/ Friends	Farmers	Media	Websites	Social Media	Supermarket
Group (ref. University	Hospital/clinic	1.31 (0.306)	1.75 (0.025) ^c^	0.716 (0.147)	0.637 (0.052)	0.835 (0.084)	1.63 (0.035)	2.13 (0.002)	3.68 (0.055)	1.07 (0.805)
	Supermarket	1.35 (0.208)	0.974 (0.903)	0.556 (0.325)	0.707 (0.084)	1.24 (0.319)	1.04 (0.832)	1.83 (0.004)	1.65 (0.043)	0.983 (0.945)
Age ^b^ (ref. up to 24 years)	50 and over	0.450 (0.002)	0.590 (0.029)	1.02 (0.801)	3.09 (0.000)	1.01 (0.798)	1.32 (0.227)	0.579 (0.022)	1.22 (0.832)	1.14 (0.634)
	25 to 49	0.574 (0.012)	0.802 (0.277)	1.00 (0.995)	2.04 (0.000)	1.20 (0.282)	1.04 (0.832)	1.00 (0.761)	1.11 (0.344)	1.03 (0.896)
Gender (ref. man)	Woman	0.916 (0.554)	0.945 (0.683)	1.53 (0.001)	0.911 (0.475)	0.836 (0.182)	0.384 (0.344)	0.799 (0.102)	0.894 (0.344)	0.758 (0.087)
Income ^d^ (ref. > 5)	Up to 5	1.30 (0.117)	1.14 (0.391)	0.968 (0.822)	0.854 (0.277)	1.70 (0.042)	1.37 (0.030)	1.43 (0.018)	1.71 (0.002)	0.912 (0.609)
Education (ref. at least college)	Up to high school	0.682 (0.038)	1.13 (0.495)	1.13 (0.486)	2.04 (0.000)	3.21 (0.000)	0.786 (0.152)	1.38 (0.069)	1.20 (0.156)	1.96 (0.000)

^a^ Government authorities and food industry were not impacted by any variable; ^b^ Logistic regression; ^c^ Hospitals/clinics against Supermarket (ref) = 1.71 (0.002); ^d^ family minimum wage; MD = medical doctor; NGOs = nongovernmental organizations.

**Table 3:** Binomial regression logistic analysis between risk perception (concern) about some hazards and trust in government and nongovernmental organizations (NGOs) in the Federal District population. Results are given as OR [LL-UP], *p*.

Worry Regarding * (High vs. Low)	Government	NGOs
Pesticides	0.694 [0.499–0.966], 0.030	6.48 [2.95–14.26], 0.000
GM food	0.503 [0.265–0.953], 0.035	3.97 [2.38–6.63], 0.000
Heavy metals	2.10 [1.08–4.05], 0.028	2.21 [1.09–4.51], 0.028

* Investigated in detail by Rembischevski et al. [25]; GM = genetically modified; OR = odds ratio [lower level-upper level at 95% confidence].

**Table 4:** Ordinal or multinomial logistic regression analysis (for media only) of trust in different sources of information and sociodemographic characteristics of Brazilian health surveillance employees. ^a,b^ Results are given as odds ratio (*p*).

Variable		Scientists/ Universities	MD/Health Professionals	Family/ Friends	Farmers	Media	Government	Websites	Social Media	Supermarket
Age (ref. 18 to 30 years)	50 and over	0.515 (0.013)	0.74 (0.186)	3.46 (0.000)	1.30 (0.51)	0.300 (0.03)	0.790 (0.277)	1.20 (0.283)	1.62 (0.086)	1.16 (0.545)
	31 to 49	0.672 (0.23) ^d^	1.30 (0.069)	1.47 (0.039) ^e^	0.829 (0.406)	1.02 (0.149)	1.45 (0.039) ^f^	1.02 (0.659)	1.14 (0.463)	1.29 (0.156)
Gender (ref. man)	Woman	0.924 (0.619)	1.00 (0.954)	0.750 (0.19)	1.05 (0.726)	0.479 (0.004)	0.734 (0.680)	0.920 (0.555)	0.845 (0.325)	1.04 (0.788)
Income ^c^ (ref. > 5)	Up to 5	0.629 (0.18)	1.85 (0.000)	1.71 (0.000)	1.53 (0.002)	1.81 (0.025)	0.684 (0.004)	1.69 (0.000)	1.80 (0.000)	1.62 (0.003)
Education (ref. at least University)	Up to high school	0.398 (0.000)	0.653 (0.098)	1.77 (0.022)	0.768 (0.299)	2.17 (0.035)	0.714 (0.35)	1.28 (0.26)	2.15 (0.004)	0.752 (0.336)

^a^ Nongovernmental organizations and food industry were not impacted by any variable; ^b^ multinominal regression, high vs. low; ^c^ family minimum wage; ^d^ 31 to 49 (ref 50 and over): OR = 1.47 (0.023); ^e^ 31 to 49 (ref 50 and over): OR = 0.437 (0.000); ^f^ 31 to 49 (ref 50 and over): OR = 1.70 (0.000). MD = medical doctors.

## Data Availability

The data presented in this study are available on request from the corresponding author.

## Supplementary Materials

The following supporting information can be downloaded at https://www.mdpi.com/article/10.3390/foods12173285/s1: Questionnaire: Risk perception, consumer behavior and trust in information sources and performance of government on food safety issues.
