# Supplementary material for: Consumers’ Trust in Different Sources of Information Related to Food Hazards and Their Judgment of Government Performance—A Cross-Sectional Study in Brazil
Source: Foods. 2023 Sep 1;12(17):3285. doi: 10.3390/foods12173285 (PMC10486621; doi:10.3390/foods12173285)
Supplement: Supplementary file 1 [file foods-12-03285-s001.zip › foods-2481250-supplementary.pdf]

Consumers' Trust in Different Sources of Information Related to Food Hazards and Their Judgment of Government Performance—A Cross-Sectional Study in Brazil

Peter Rembischevski 1 and Eloisa Dutra Caldas 2,*

1 Brazilian Health Regulatory Agency (Anvisa), Brasilia 71205-050, DF, Brazil; peter.rembischevski@anvisa.gov.br

2 Laboratory of Toxicology, Department of Pharmacy, Faculty of Health Sciences, University of Brasilia, Darcy Ribeiro Campus, Brasilia 70910-900, DF, Brazil

* Correspondence: eloisa@unb.br

SUPPLEMENTARY MATERIAL

Questionnaire

Risk perception, consumer behavior and trust in information sources and performance of government on food safety issues

Participant number: ______
Location: ______________________________
Gender: Male ( ) Female ( ) Other ( )
Age: _____________

1. Monthly family income:
( ) Up to R$998 (1 minimum wage)
( ) Between R$998 and R$2.000
( ) Between R$2.001 and R$ 5.000
( ) Between R$5.001 and R$ 10.000
( ) Over R$10.000

2. Education:
( ) Incomplete primary school
( ) Complete primary school
( ) Incomplete high school
( ) Complete high school
( ) Incomplete college
( ) Complete college
( ) Postgraduate

3. How much do you consume of foods below? 1: not much; 2: medium or 3: a lot.
Canned and industrialized -
Fruits and vegetables -
Meat and eggs -
Cereals and grains - (eg., rice, bean)
Carbohydrates (eg., pasta, bread) -

4. What is the main characteristic do you seek in the food? You can tick more than one.
( ) Tasty
( ) Nutritious
( ) Safe
( ) Healthy
( ) Low price

5. Do you think food can be deleterious to health?
( ) Often
( ) Occasionally
( ) Rarely
( ) Never

6. What is your worry level about the presence of chemicals or contaminants in food?
( ) High
( ) Medium
( ) Low
( ) None

7. When was the last time you´ve read, saw or heard that foods can be harmful to health due to the presence of chemicals?
( ) In the last 7 days
( ) About 1 month ago
( ) In the last year
( ) More than 1 year ago
( ) Never/I don't remember

8. Do you read food labels?
( ) Always
( ) Almost always
( ) Sometimes
( ) I'm not used to
( ) The expiry date only

9. If you are not used to read food labels, point out the main reason:
( ) Too small letters
( ) Technical terms, I don't understand
( ) I have no interest
( ) I don't have the time or patience
( ) Others: __________________

10. Have you ever stopped eating something you like for fear of not being good for your health?
( ) More than once
( ) Once
( ) Never
( ) I don’t remember

11. Do you use to buy organic food?
( ) Exclusively
( ) Almost always
( ) Sometimes
( ) Never, I don't think it's relevant

( ) I would like it, but it's expensive
( ) I do not know what this means

12. Do you believe it is possible to produce food without pesticides in enough quantity for the whole population?

( ) Yes, all foods
( ) Yes, some foods
( ) Maybe, I am not sure
( ) No food
( ) I have no opinion about

13. Do you think it´s relevant for some foods to have the presence of pesticides indicated in the label?

( ) Yes, at least inform if it contains or not
( ) Yes, and inform all pesticides
( ) I see no need
( ) I have no opinion about

14. What do you think pesticides present in food can cause to health? You can tick more than option.

( ) Cancer
( ) Hormonal effects
( ) Reproductive effects
( ) Effects on the brain/nervous system
( ) Headache, malaise, nausea
( ) Other effects
( ) No negative health effects
( ) I don't know/I prefer not to give an opinion

15. Have you ever had any symptoms or illnesses that you believed was caused by pesticides or chemical contaminants in food?

( ) Never
( ) More than once
( ) Once
( ) Maybe, I am not sure

16. Do you make any procedure at home to remove or reduce pesticide levels in food, such as washing and peeling?

( ) Always
( ) Often
( ) Rarely
( ) Never

17. Dou you know the meaning of the T symbol?

( ) Yes
( ) Maybe
( ) No, I´ve never notice it.

18) Do you refuse buying food with this symbol?

( ) Always/almost always
( ) Sometimes
( ) Not relevant.

19. How do you judge the performance of government institutions responsible for guarantee food safety? (Anvisa, Ministry of Agriculture, Ministry of Health)

( ) Very good
( ) Good
( ) Acceptable
( ) Low
( ) Very low
( ) Don´t have an opinion

20. If you considered the performance low or very low, what could be done to improve it?

( ) Increase inspection in industries
( ) Increase inspection in commerce
( ) Apply more fines to offenders
( ) Improve risk communication
( ) Prioritize consumer health
( ) Require more information on the label

21. When was the last time you´ve read, saw or heard that genetically modified food may be harmful to thealth?

( ) In the last 7 days
( ) About 1 month ago
( ) Between 1 and 6 months
( ) More than 6 months ago
( ) Never/I don't remember

22. From the list of substances or technologies associated to food, chose the best option that reflects your worry level regarding their impact to health

**Substance or technology**	**Don´t know**	**Slightly/not worried at all**	**Moderately worried**	**Very worry**
Sugar				
Salt				
*Agrótoxico**				
Pesticide				
Plant protection product				
Food additive (eg. sweetener, dyes, preserving)				
Heavy metal (eg. mercury, lead, arsenic)				
Mycotoxins (eg. aflatoxins)				
Hormones and antibiotics (in meat)				
Substances present in food packaging (eg. bisphenol A, phthalates)				
Transgenic/genetically modified food				
Animal cloning				
Nanotechnology				

* legal term for pesticides in Brazil

23. From the list of source of information about the risks from the presence of chemicals in food, chose the best option that reflect your trust level

**Source of information**	**Don't know/don't have an opinion**	**Low/don´t trust**	**Trust reasonably**	**Trust a lot**
Media (TV, radio, journal)				
websites/blogs				
Social media (eg. Facebook, Twitter)				
Scientists/universities				
government authorities				
Non-governmental organizations				
Food industry				
Supermarket				
Medical doctor (MD)/health professionals				
Farmers				
Family and friends				
